# Targeting the ATP Synthase in *Staphylococcus aureus* Small Colony Variants, *Streptococcus pyogenes* and Pathogenic Fungi

**DOI:** 10.3390/antibiotics10040376

**Published:** 2021-04-02

**Authors:** Martin Vestergaard, Sahar Roshanak, Hanne Ingmer

**Affiliations:** 1 Department of Veterinary and Animal Sciences, Faculty of Health and Medical Sciences, University of Copenhagen, Stigbøjlen 4, DK-1870 Frederiksberg C, Denmark; mave@sund.ku.dk (M.V.); s.roshanak@mail.um.ac.ir (S.R.); 2 Department of Food Science and Technology, Faculty of Agriculture, Ferdowsi University of Mashhad, Mashhad 9177948974, Iran

**Keywords:** ATP synthase, oligomycin A, DCCD, resveratrol, *Staphylococcus aureus*, small colony variants, SCV, *Streptococcus* pyogenes, *Candida albicans*

## Abstract

The ATP synthase has been validated as a druggable target with the approval of the ATP synthase inhibitor, bedaquiline, for treatment of drug-resistant *Mycobacterium tuberculosis*, a bacterial species in which the ATP synthase is essential for viability. Gene inactivation studies have also shown that the ATP synthase is essential among Streptococci, and some studies even suggest that inhibition of the ATP synthase is a strategy for the elimination of *Staphylococcus aureus* small colony variants with deficiencies in the electron transport chain, as well as pathogenic fungi, such as *Candida albicans*. Here we investigated five structurally diverse ATP synthase inhibitors, namely *N*,*N*′-dicyclohexylcarbodiimide (DCCD), oligomycin A, tomatidine, resveratrol and piceatannol, for their growth inhibitory activity against the bacterial strains *Streptococcus pyogenes*, *S. aureus* and two isogenic small colony variants, as well as the pathogenic fungal species, *C. albicans* and *Aspergillus niger*. DCCD showed broad-spectrum inhibitory activity against all the strains (minimum inhibitory concentration (MIC) 2–16 µg/mL), except for *S. aureus*, where the ATP synthase is dispensable for growth. Contrarily, oligomycin A selectively inhibited the fungal strains (MIC 1–8 µg/mL), while tomatidine showed very potent, but selective, activity against small colony variants of *S. aureus* with compromised electron transport chain activity (MIC 0.0625 µg/mL). Small colony variants of *S. aureus* were also more sensitive to resveratrol and piceatannol than the wild-type strain, and piceatannol inhibited *S. pyogenes* at 16–32 µg/mL. We previously showed that transposon inactivation of the ATP synthase sensitizes *S. aureus* towards polymyxin B and colistin, and here we demonstrate that treatment with structurally diverse ATP synthase inhibitors sensitized *S. aureus* towards polymyxin B. Collectively, our data show that ATP synthase inhibitors can have selective inhibitory activity against pathogenic microorganisms in which the ATP synthase is essential. The data also show that the inhibition of the ATP synthase in *Streptococcus pyogenes* may be a new strategy for development of a narrow-spectrum antibiotic class. In other major bacterial pathogens, such as *S. aureus* and potentially *Escherichia coli*, where the ATP synthase is dispensable, the ATP synthase inhibitors may be applied in combination with antimicrobial peptides to provide new therapeutic options.

## 1. Introduction

The majority of the clinically available antibiotics interfere with a limited set of targets, including inhibition of cell wall biosynthesis, protein synthesis, DNA synthesis, RNA synthesis, folic acid metabolism and disruption of cell membrane integrity [1]. In the past decade, targeting bacterial energetics has been added to the list with the approval of the anti-mycobacterial antibiotic, bedaquiline, which targets the membrane-bound F_1_F_o_-ATP synthase [2]. ATP synthesis by the membrane-bound ATP synthase is essential for both growing and nongrowing mycobacteria, including *Mycobacterium tuberculosis* [3] and *Mycobacterium smegmatis* [4]. Despite resemblance to the human mitochondrial ATP synthase, bedaquiline selectively inhibits the ATP synthase of *Mycobacterial* spp. [5].

The ATP synthase is also essential in Streptococci, where all eight genes encoding for subunits of the ATP synthase in *Streptococcus sanguinis* were identified as being essential in a genome-wide screen [6]. Three of the ATP synthase genes were subsequently confirmed to be essential in *Streptococcus pneumoniae* and *Streptococcus mutants* [6]. Furthermore, essentiality of the ATP synthase has been confirmed by others in *S. pneumoniae* [7,8] and *Streptococcus pyogenes* [9]. Streptococci lack a respiratory chain to establish the proton motive force and the ATP synthase is indispensable for establishing the proton motive force needed for various transport processes and to maintain pH homeostasis [6].

In contrast, the ATP synthase is not essential in the facultative anaerobic pathogen *Staphylococcus aureus* [10]. However, the ATP synthase was recently established as the target of tomatidine, which selectively inhibits growth of small colony variants with a compromised electron transport chain (ETC-SCVs), while having no inhibitory activity against wild-type cells [11]. ETC-SCVs often carry mutations in genes that contribute to the biosynthesis of the electron carrier menaquinone or the heme group in cytochromes [12]. Even though the ATP synthase is not essential for viability in *S. aureus*, the genetic inactivation of subunits of the ATP synthase leads to increased susceptibility towards different cationic antimicrobials, including aminoglycosides [13,14], polymyxins [15] and certain human antimicrobial peptides [16].

Besides tomatidine, multiple ATP synthase inhibitors have been identified, such as *N*,*N*′-dicyclohexylcarbodiimide (DCCD) and oligomycin A, which non-selectively inhibits ATP synthases in *S. aureus*, *Escherichia coli* and human mitochondria [17]. Furthermore, the polyphenolic compounds, resveratrol and piceatannol, inhibit the ATP synthase in *E. coli* [18]. Consistent with the genetic studies, the ATP synthase inhibitors tomatidine [19], DCCD [20] and resveratrol [21] sensitize *S. aureus* towards aminoglycosides, while resveratrol has been shown to sensitize *S*. *aureus* to polymyxin B [16].

The ATP synthase consists of multiple subunits, with an overall assembly of a membrane-embedded F_0_-domain and a cytoplasmic F_1_-domain. The composition of bacterial ATP synthases generally consists of the subunits α_3_β_3_γδε, constituting the F_1_-domain and the F_0_-domain that comprises the ab_2_c_9–15_ subunits [22]. Proton translocation across the lipid bilayer occurs at the F_0_ *c*-ring, an oligomer of the subunit *c*, which is encoded by *atpE* in bacteria [23].

Oligomycin A was originally discovered as an antifungal in 1954 [24] and its binding to the yeast mitochondrial ATP synthase subunit *c*-ring has been elucidated [25]. Oligomycin A effectively inhibits a range of pathogenic fungal species, including *Candida albicans* and *Aspergillus niger* [26], suggesting that the ATP synthase could also be an essential target in some pathogenic fungi.

In the present study, we investigated the impact of structurally diverse ATP synthase inhibitors on a wide range of microorganisms, namely *Streptococcus pyogenes*, *Candida albicans* and *Aspergillus niger*, as well as towards the *S. aureus* ETC-SCVs (*menD*::Tn and *hemB*::Tn mutants [10]). Our results point to interesting, novel therapeutic possibilities and suggest the ATP synthase may be a druggable target in many organisms.

## 2. Results and Discussion

### 2.1. DCCD Displays Broad-Spectrum Inhibitory Activity

The ATP synthase inhibitor DCCD showed broad-spectrum activity and inhibited growth of both the *S. aureus* ETC-SCVs, *S. pyogenes* and all fungal isolates (minimum inhibitory concentration (MIC) 2–16 µg/mL) (Table 1). DCCD did not inhibit the growth of the wild-type *S. aureus*, in which the ATP synthase is dispensable for growth [10]. Even though DCCD did not inhibit the growth of wild-type *S. aureus* (MIC > 128 µg/mL), at a subinhibitory concentration (16 µg/mL), this compound sensitized *S. aureus* towards polymyxin B (Table 2).

**Figure 1:**
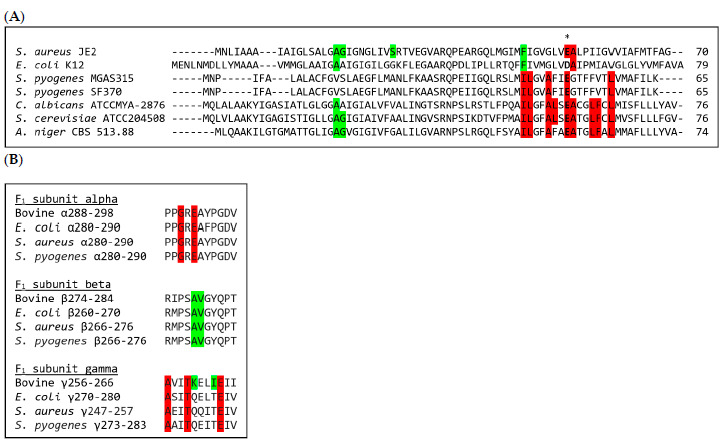
Amino acid sequence alignments of four ATP synthase subunits involved in the binding of tested ATP synthase inhibitors. (**A**) Amino acid sequence alignments of the F_0_ subunit *c* for bacterial and fungal isolates. The essential carboxyl-containing amino acid (glutamic acid (E) or aspartic acid (D)) for proton translocation is indicated with the asterisk and bold letters. Amino acids that upon mutation in *S. aureus* conferred resistance to tomatidine are marked in green [11], along with shared amino acids in the other microorganisms. The amino acid residues constituting the binding site of oligomycin A in *S. cerevisiae* are marked in red [25], along with shared amino acids in the other microorganisms. (**B**) Amino acid sequence alignments of short regions of the ATP synthase subunits α, β and γ involved in the binding of resveratrol to the F_1_-domain of the bovine ATP synthase [34], with corresponding regions of *E. coli* as previously aligned [18]. Here, alignments of corresponding sequences for *S. aureus* and *S. pyogenes* are added. Residues marked in green are involved in hydrophobic interactions with resveratrol/piceatannol, while residues marked in red provide additional nonpolar interactions [34]. The residues involved in resveratrol binding are identical between the *E. coli*, *S. aureus* and *S. pyogenes* sequences.

DCCD reacts covalently with carboxylic groups in both the F_1_ β-subunit and F_0_ subunit *c* [27]. In *E. coli*, DCCD reacts with the carboxylic group in aspartic acid at position 61 in the F_0_ subunit *c* [27], while this residue in the organisms that we tested was substituted with the carboxyl-containing amino acid, glutamic acid (Figure 1A, the position is marked with an asterisk and bold letters). This carboxylic group is directly involved in proton translocation and its modification with DCCD abolishes the function of F_0_ [28]. The carboxyl-containing amino acids in the subunit *c* are well conserved in bacterial, mitochondrial and chloroplast enzymes [11,29], indicating that DCCD could be a broad-spectrum ATP synthase inhibitor.

Even though DCCD non-selectively inhibits ATP synthases in human mitochondria, *S. aureus* and *E. coli* [11,17], which precludes it from clinical use, it remains a valuable tool for initial investigations on the implications of ATP synthase inhibition in various organisms.

### 2.2. Oligomycin A Selectively Inhibits Fungal Pathogens

Oligomycin A selectively inhibited the growth of the fungal pathogens at concentrations ranging from 1 to 8 µg/mL, while showing no inhibitory activity against the *S. aureus* ETC-SCVs nor *S. pyogenes*.

The oligomycin binding site was determined in the yeast *Saccharomyces cerevisiae* and involves interactions with nine amino acids [25]. The binding site spanned the carboxyl-containing amino acid of the subunit *c* of F_0_, namely Glu59, corresponding to Asp61 in *E. coli*. The amino acids of the *S. cerevisiae* subunit *c* involved in binding of oligomycin are highlighted in red in Figure 1A. The amino acids were completely conserved between *S. cerevisiae* and *C. albicans*, while a single amino acid was changed in the *A. niger* protein sequence. Only few of the residues were conserved in the bacterial isolates, which may explain the lack of inhibitory activity against *S. pyogenes* and *S. aureus* ETC-SCVs.

Even though oligomycin A had no inhibitory activity against *S. aureus* ETC-SCVs, oligomycin A still sensitized wild-type *S. aureus* to polymyxin B.

### 2.3. Tomatidine Is Highly Selective for S. aureus Small Colony Variants

Tomatidine was the most potent inhibitor against *S. aureus* ETC-SCVs, with the MIC being 0.0625 µg/mL, without having inhibitory activity against the wild-type *S. aureus* (Table 1), corroborating previous results of Mitchell and colleagues [30]. Contrarily, tomatidine did not inhibit growth of *S. pyogenes* nor the fungal strains (Table 1), which supports the observation that tomatidine primarily inhibits members of the order *Bacillales*, which includes pathogens such as *S. aureus*, *Listeria monocytogenes* and *Bacillus anthrasis* [31].

Tomatidine was also shown to target the F_0_ subunit *c* [11]. The exact binding site was not determined; however, resistance-conferring mutations were identified (Figure 1A, green markings highlight amino acids that upon mutation confer resistance to tomatidine in *S. aureus* ETC-SCVs [11]). Members of the order *Bacillales* show highly similar F_0_ subunit *c* sequences, and it was hypothesized by Boulet and colleagues that, for example, *Streptococcus pneumoniae* is not targeted by tomatidine because of low sequence identity of the target F_0_ subunit *c* between *S. aureus* and *S. pneumoniae* [11].

In Figure 1A, we show that the sequence similarity of F_0_ subunit *c* between *S. aureus* and *S. pyogenes*, *C. albicans* or *A. niger* is also low (31%–37% amino acid identity), which may also explain the lack of inhibitory activity of tomatidine against these strains.

Tomatidine also sensitized wild-type *S. aureus* to polymyxin B, similar to the other ATP synthase inhibitors, DCCD and oligomycin A, showing that structurally diverse ATP synthase inhibitors in general sensitize *S. aureus* to polymxin B.

### 2.4. Resveratrol and Piceatannol Inhibits S. aureus Small Colony Variants and S. pyogenes

Resveratrol and its hydroxylated analogue piceatannol, were 2–8 times more active against the *S. aureus* ETC-SCVs and *S. pyogenes* than the wild-type *S. aureus*. Piceatannol inhibited *S. pyogenes* at 16–32 µg/mL, while resveratrol and piceatannol did not prevent the growth of the fungal strains at the tested concentrations (MIC > 32 µg/mL). Resveratrol has been widely studied for its antibacterial and antifungal properties [32] and others have shown similar levels of inhibition of *S. pyogenes* by resveratrol [33]. Piceatannol is less studied for its antimicrobial properties than resveratrol; however, our data suggest that piceatannol is more potent than resveratrol, at least against *S. pyogenes* and *S. aureus* ETC-SCVs.

Resveratrol and piceatannol were determined to bind to the F_1_-domain in the bovine ATP synthase in a pocket between a β-, α- and the γ-subunit [34]. The residues involved in the interaction with resveratrol and piceatannol are highlighted in Figure 1B [34]. Resveratrol was also shown to inhibit the ATP synthase in *E. coli* and six residues of the eight residues involved in the interaction are conserved between the bovine and the *E. coli* sequence, suggesting a similar binding pocket in the *E. coli* F_1_-domain [18]. The residues that are important for the resveratrol/piceatannol interaction with the bovine F_1_-domain, and hypothesized to constitute a similar binding site in *E. coli* [18], are identical between *E. coli*, *S. aureus* and *S. pyogenes* (Figure 1), suggesting a similar binding pocket in the Gram-positive pathogens as in *E. coli*.

## 3. Conclusions

In this study, we showed that structurally diverse ATP synthase inhibitors display variability in the inhibitory activity against bacterial and fungal species, in which the ATP synthase is indispensable for growth. The ATP synthase inhibitor, DCCD, had the broadest spectrum of activity compared with the other ATP synthase inhibitors investigated. Tomatidine was the most active against *S. aureus* ETC-SCVs, while oligomycin A was the most potent antifungal inhibitor. The ATP synthase is essential in Streptococci [6,7,8,9], and we showed that resveratrol and piceatannol display intermediary inhibitory activity against two *S. pyogenes* isolates (Table 1). Others have also identified inhibitors of the ATP synthase to inhibit the growth of *S. pneumoniae* [17,35] and other Streptococcal species [35]. Collectively, these data suggest that the ATP synthase in Streptococcal species is a druggable target for novel antibiotics with the potential for narrow-spectrum activity.

Our study also supports the accumulating evidence that the ATP synthase can be targeted to sensitize *S. aureus* to polymyxins, and here we showed that structurally diverse ATP synthase inhibitors all worked as polymyxin B sensitizers (Table 2), corroborating previous studies with the ATP synthase inhibitors venturicidin A [36] and resveratrol [16]. Recent reports show that resveratrol can also sensitize several Gram-negative species, including *E. coli* and *K. pneumoniae*, to colistin; however, the mechanism of potentiation was not investigated in these studies [37,38]. It remains to be explored if, in general, other ATP synthase inhibitors sensitize Gram-negative bacteria to polymyxins, where this antibiotic class is considered as last-resort antibiotics [39].

## 4. Materials and Methods

### 4.1. Bacterial Strains, Growth Conditions and Chemicals

The bacterial strains used in this study are highlighted in Table 1. Chemicals used in this study include tomatidine hydrochloride (Merck KGaA, Darmstadt, Germany), oligomycin A (Santa Cruz Biotechnology, Dallas, TX, USA), *N*,*N*′-Dicyclohexylcarbodiimide (Merck KGaA, Darmstadt, Germany), piceatannol (Santa Cruz Biotechnology, Dallas, TX, USA) and resveratrol (Santa Cruz Biotechnology, Dallas, TX, USA).

All bacterial strains and the two *Candida albicans* isolates were routinely cultured at 37 °C in tryptic soy broth (TSB) or on tryptic soy agar (TSA), whereas *Aspergillus niger* was cultured in nutrient broth (NB).

### 4.2. Minimum Inhibitory Concentration

The minimum inhibitory concentrations (MICs) for the ATP synthase inhibitors were determined using the twofold broth microdilution assay.

### 4.3. Bacteria

Overnight cultures of the bacterial isolates were diluted in physiological saline (0.9% NaCl) to reach a turbidity of 0.5 McFarland (Sensititre^®^ nephelometer and the Sensititre^®^ McFarland Standard). Bacterial suspensions were adjusted to 5 × 10^5^ CFU/mL in TSB broth containing twofold dilutions of the inhibitors in a final volume of 100 µL. The plates were incubated for 24 h at 37 °C without shaking. MIC was defined as the concentration of the agents that completely prevented visible growth. All experiments were performed with biological triplicates.

### 4.4. Fungi

From the agar plates, *A. niger* and *C. albicans* were diluted in physiological saline (0.9% NaCl) to reach an initial cell density of 10^6^ CFU/mL. Fungal suspensions were adjusted to 1 × 10^5^ CFU/mL in NB (*A. niger*) or TSB (*C. albicans*) solutions containing twofold dilutions of the inhibitors in a final volume of 100 µl. The plates were incubated for 48 h at 30 °C without shaking. MIC was defined as the concentration of the agents that completely prevented visible growth. All experiments were performed with biological triplicates.

Growth controls with the solvents (DMSO and ethanol) of the ATP synthase inhibitors were performed at the highest concentration (3.2%) that the microorganisms were exposed to.

### 4.5. Combination of ATP Synthase Inhibitors and Polymyxin B

The MIC for polymyxin B was determined using Etest (bioMérieux, France) on TSA plates in the absence and in the presence of ATP synthase inhibitors (DCCD, oligomycin A and tomatidine) supplemented at a single subinhibitory concentration (16 µg/mL). From overnight cultures, strains were diluted to approximately 10^8^ CFU/mL and then distributed on TSA plates using a sterile cotton swab. MIC was determined upon incubation at 37 °C for 24 h. All experiments were performed with biological triplicates.

### 4.6. Protein Sequence Alignment of ATP Synthase Subunits

Protein sequences of the c-, γ-, α- and β-subunits from *S. aureus* JE2, *S. pyogenes* MGAS315, *S. pyogenes* SF370, *E. coli* K12, *C. albicans* ATCCMYA-2876 (sequences of tested strains could not be retrieved), *A. niger* CBS 513.88 (sequences of tested strain could not be retrieved), *Saccharamyces cerevisiae* ATCC204508 and *Bos Taurus* were retrieved from the KEGG GENOME Database. Multiple sequence alignment was conducted with Clustal Omega [40].

## Figures and Tables

**Table 1:** Minimum inhibitory concentrations of ATP synthase inhibitors against *S. aureus* small colony variants, *S. pyogenes* and the fungal pathogens *C. albicans* and *A. niger*.

	MIC (µg/mL)				
	Tomatidine	Oligomycin A	DCCD	Piceatannol	Resveratrol
*S. aureus* JE2	>128	>128	>128	128	256
*S. aureus* JE2 *menD*::Tn [10]	0.0625	>128	2–4	32	128
*S. aureus* JE2 *hemB*::Tn [10]	0.0625	>128	4–8	64	128
*S. pyogenes* SF370	>128	>128	8–16	16–32	64
*S. pyogenes* MGAS315	>128	>128	8	16	64
*C. albicans* ATCC 64548	>32	4–8	4–8	>32	>32
*C. albicans* ATCC 64550	>32	4–8	4–8	>32	>32
*A. niger* IBT 28144	>32	1–2	16	>32	>32

**Table 2:** ATP synthase inhibitors sensitizes *S. aureus* JE2 to polymyxin B (PMB). Minimum inhibitory concentration (MIC) in µg/mL. TSA: tryptic soy agar.

Supplementation in Plate	PMB MIC (Fold Reduction)
TSA	128
TSA + 16 µg/mL Oligomycin A	32 (4)
TSA + 16 µg/mL Tomatidine	16–24 (8)
TSA + 16 µg/mL DCCD	48 (2.6)

## Data Availability

The data presented in this study are available on request from the corresponding author.
